# TMEM115: a promising marker for glioma immunotherapy and prognosis

**DOI:** 10.3389/fimmu.2025.1598499

**Published:** 2025-06-09

**Authors:** Hang Yin, Feng Wang, Haiyan Xu, Manyu Xu, Pengpeng Lu, Xiaojing Zhang, Lei Yang, Bing Lu, Pingping Sun, Jianfei Huang

**Affiliations:** ^1^ Department of Clinical and Translational Research Center, Affiliated Hospital of Nantong University, Nantong, Jiangsu, China; ^2^ Department of Pathology, Medical School of Nantong University, Nantong, Jiangsu, China; ^3^ Department of Oncology, Medical School of Nantong University, Nantong, Jiangsu, China; ^4^ Institute of Oncology, Affiliated Hospital of Nantong University, Nantong, Jiangsu, China

**Keywords:** glioma, TMEM115, prognosis, M2 macrophage, PD-L1

## Abstract

**Background:**

Glioma is a common malignant primary brain tumor characterized by a highly immunosuppressive tumor microenvironment and unfavorable prognosis. Transmembrane protein 115 (TMEM115) is a protein-coding gene. It may play a role in the retrograde transport of proteins from the Golgi to the endoplasmic reticulum. At the same time, it may also play an indirect role in protein glycosylation at the Golgi. However, the role of TMEM115 in glioma progression is still unclear and, therefore, needs further exploration.

**Methods:**

RNA-seq data from The Cancer Genome Atlas and Chinese Glioma Genome Atlas databases of glioma patients and multiplex Immunohistochemistry by glioma tissue microarrays were analyzed to determine the expression and localization of TMEM115. Functional assessment of TMEM115 involved cell proliferation, migration, and invasion assays. Pearson’s test was utilized to evaluate the association between TMEM115 protein levels, tumor immune infiltrating cells, and immune checkpoints. In addition, χ^2^ test and Cox regression analyses were performed to investigate whether TMEM115 protein expression is related to clinical characteristics and patient outcomes.

**Results:**

The results revealed a significant increase in TMEM115 expression in tumors than in non-tumor brain tissues, and knockdown of TMEM115 affects the ability of glioma cell lines to proliferate, migrate, and invade. Additionally, a significant correlation between TMEM115 protein, M2 macrophages (CD68^+^CD163^+^), and programmed cell death ligand-1 (PD-L1). Further analysis confirmed a correlation between TMEM115 protein expression in glioma tissues, World Health Organization Grade, and patients’ poorer prognosis.

**Conclusion:**

These findings support TMEM115 as a potential independent prognostic biomarker in glioma and suggest its promise as a target for immunotherapy.

## 1 Introduction

Globally, the prevalence of glioma has been progressively increasing, and the five-year survival rate remains dismally low (1). Gliomas are classified by the World Health Organization (WHO) into four grades based on their severity, with higher grades indicating more aggressive tumors and poorer prognosis (2). The current standard treatment for glioma involves surgical resection, temozolomide chemotherapy, and radiotherapy (3). However, these treatments often fail to halt cancer progression, leading to inevitable relapse (4). Hence, there is a pressing need to identify more effective therapeutic targets (5). The tumor microenvironment (TME) plays an important role in tumor development and significantly influences cancer patients’ prognosis (6). Among the components of the TME, tumor-associated macrophages (TAMs) are the most prevalent immune cells and are central to tumor-associated inflammation. They typically promote tumor growth and metastasis and are associated with a poor prognosis (7). Additionally, the presence of the TME may render patients treated with immune checkpoint inhibitors, such as programmed cell death ligand-1 (PD-L1) (8), resistant or prone to relapse (9–11). The Cancer Genome Atlas (TCGA) provides a comprehensive repository of clinical and molecular phenotypic data from over ten thousand tumor patients across 33 kinds of tumors and represents an invaluable resource for researchers to perform extensive biological data analysis (12). The Chinese Glioma Genome Atlas (CGGA) database includes approximately 2,000 primary and recurrent glioma samples and detailed clinical information, providing easy and quick access to data resources for biological research and clinical applications (13). Our preliminary analysis utilizing the TCGA database revealed a significant increase in Transmembrane protein 115 (TMEM115) mRNA expression in glioma tissues regarding non-glioma samples, with implications for patient outcomes. In addition, the correlation between TMEM115 and clinicopathological features was analyzed using two public databases, TCGA and CGGA. TMEM115, located in the Golgi cisterna membrane and involved in glycosylation (14). More importantly, our group has found TMEM115 to be a promising prognostic indicator for Hepatocellular Carcinoma in predicting response to immunotherapy in previous studies (15). Although previous studies have reported elevated mRNA levels of TMEM115 in glioma compared to benign tissues, with implications for poorer patient survival, current literature indicates a poor correlation between mRNA and protein levels, and their expression can be inconsistent (16).

In oncological studies, multiplex Immunohistochemistry (mIHC) is a vital technique that employs multiple antibodies to precisely recognize specific cell types within tumor tissues and to analyze the two-dimensional spatial relationship between these cells and cancer cells. The primary obstacle in mIHC is extracting effective spatial distribution features from high-resolution microscopic images, which contain numerous pixels with intensity and color information (17, 18). Automated methods based on machine learning (ML) are of great significance in this field. ML methods can be fully automated or semi-supervised. For instance, Akoya’s InForm software package, which incorporates user-defined region segmentation to enhance tissue or cell classification, is a case in point. By utilizing the pattern-recognition capabilities of ML algorithms, these methods can extract valuable information from complex image data, thus offering novel perspectives and tools for oncology research (17, 19).

In this study, we employed multicolor mIHC and InForm to assess TMEM115 protein expression within the TME based on glioma tissue microarrays (TMAs) alongside patients’ clinical data. Furthermore, we explored its relationship with immune checkpoints, particularly PD-L1, integrating clinical and immunotherapeutic approaches. Additionally, we investigated the association between TMEM115 and clinicopathological features to elucidate its prognostic significance for five-year survival.

## 2 Materials and methods

### 2.1 Glioma data acquisition

We utilized UCSC XENA (https://xenabrowser.net/datapages/) to analyze processed RNA-seq data from TCGA (https://portal.gdc.cancer.gov) and Genotype-Tissue Expression (GTEx) database (https://www.gtexportal.org/home/), which were uniformly processed using Toil methods, for comparing the mRNA levels of TMEM115 in glioma (n = 689) and non-tumor tissues (n = 1157). Additionally, clinical data were retrieved to investigate whether TMEM115 expression is linked to glioma patients’ Overall Survival (OS) and Disease-Free Survival (DFS). In addition, we extracted data and corresponding clinical information from the GBM and LGG datasets of the TCGA database as well as from the mRNAseq_325 (CGGA 325) and mRNAseq_693 (CGGA 693) datasets of the CGGA database (http://www.cgga.org.cn) for clinical characterization.

### 2.2 Clinical samples and information

TMAs comprising 214 tissue samples were collected from the Nantong University Affiliated Hospital between 2013 and 2017 (16). These samples included 189 glioma tissues and 25 non-tumor brain tissues from individuals with benign brain diseases. Patient data, including gender, age, histological classification, molecular classification, WHO grading, and whether they had undergone radiotherapy or chemotherapy, were collected according to the 2021 WHO Classification of Tumors of the Central Nervous System (20). This study was approved by the ethics committee of our hospital (Grant No. 2018-K020).

### 2.3 Fluorescence-based mIHC staining

The Opal 7-color manual IHC kit (NEL797B001KT; PerkinElmer, MA, US) was used for mIHC staining as previously described (15, 21). Briefly, tissue slices were dewaxed and hydrated, followed by heat-induced antigen retrieval using an AR6 solution (AR600, PerkinElmer) in a microwave oven. Next, the slides were incubated with indicated primary antibodies, followed by the polymer HRP-conjugated secondary antibody (ARH100EA, AKOYA). Fluorescent dyes were added for signal visualization, and multiple staining was accomplished by repeating the process with microwave heating treatments performed between each step. Lastly, FluoroshieldTM with DAPI (F6057, Sigma, NY, USA) was utilized to stain the nucleus for visualization. In this experiment, the following antibodies were used: anti-TMEM115 antibody (1:5000, NBP1-80898, Novus, USA), anti-CD68 antibody (1:1500, 76437S, Cell Signaling Technology, USA), anti-CD163 antibody (1:200, 93498S, Cell Signaling Technology, USA), anti-CD86 antibody (1:500, 91882S, Cell Signaling Technology, USA), anti-PD-L1 antibody (1:500, 13684S, Cell Signaling Technology, USA).

### 2.4 Machine learning scoring

We utilized an automated imaging system (Vectra 3.0, Perkin Elmer/Akoya, USA) to scan and quantify all samples, followed by analysis and scoring using InForm software (Akoya, USA). The fluorescence intensity threshold of each marker used in the analysis was assessed to confirm the presence of a specific cell phenotype. The final score (range: 0-100) was determined based on the ratio of positively stained cells to nuclei times 100%.

### 2.5 Cell clustering

Cell lines (U87MG, U251, and HEK293T) were obtained from the Cell Bank/Stem Cell Bank at the Chinese Academy of Sciences (Shanghai, China). They were cultured in a complete growth medium consisting of the appropriate base medium (Gibco, Grand Island, NY, USA), supplemented with 10% fetal bovine serum (Shuangru, Jiangsu, China), and 1% penicillin-streptomycin (NCM Biotech, Jiangsu, China). Additionally, U251 required the inclusion of Non-Essential Amino Acids Solution (C0332; Beyotime) and Sodium Pyruvate (C0331; Beyotime). The cells were incubated at 37°C with 5% carbon dioxide in an incubator.

### 2.6 Plasmid construction, lentivirus, and cell transfection

On Merck’s website (https://www.sigmaaldrich.cn/CN/zh/semi-configurators/shrna?activeLink=productSearch), designing two different short hairpin RNA (shRNA) sequences and a targeting TMEM115 control sequence (shRNA negative control). They were synthesized by the company of Tsingke Biotechnology (Jiangsu, China). The shRNA forward sequences targeting TMEM115 were shRNA (NC): (TTCTCCGAACGTGTCACGT) shRNA (TMEM115 1^#^) (GCCCTCATCTGAGGTAAGAAT) and shRNA (TMEM115 2^#^) (GAAGGTAAAGATATGCCAGAA). After annealing and purification, the fragments were integrated into the pLV-shRNA vector, reseparately. This vector was co-transfected with pMD2.G and psPAX2 plasmids into HEK293T cells to generate recombinant lentiviral particles. After 72 hours, the supernatant containing lentiviral particles was collected and infected with glioma cell lines. After appropriate culture screening, target cells were collected and analyzed by Western blotting to verify transfection efficiency, followed by cell proliferation, migration, and invasion assays.

### 2.7 Western blot

Total proteins from glioma cell samples were extracted using RIPA lysis buffer (p0013B; Beyotime) with protease and phosphatase inhibitors (P1260; Solarbio, Beijing, China). Equal amounts of proteins were added to a 10% SDS-PAGE gel (PG112; Epizyme Biotech, Shanghai, China) for electrophoretic separation and transferred to a 0.45µm PVDF membrane (Millipore, USA). Afterward, the membrane was blocked with 5% skim milk and incubated overnight with primary antibodies such as TMEM115 antibody (1:1000, 25536-1-AP, protein tech) or β-Actin antibody (1:5000, AC026, ABclonal). The next day, it was detected again with Peroxidase-conjugated AffiniPure Goat Anti-Rabbit IgG (H+L) antibody (1:10000, 111-035-003, Jackson ImmunoResearch). Protein bands were visualized using an ECL kit (BL520B; Biosharp, Beijing, China) and captured by a chemiluminescence imaging system (Tanon 4600SF, Nanjing, China). Finally, optical density was calculated using Image J software to determine the TMEM115 protein expression level.

### 2.8 Cell proliferation analysis

2× 10^3^ U87MG and U251 cells were seeded into 96-well plates, and after the cells were attached to the wall, the medium was discarded, and the cells were treated according to the instructions of Cell Counting Kit-8 (CCK-8) (C6050; NCM Biotech, Suzhou, China) reagent, and the absorbance was detected after incubating the cells at 37°C for 2h to reflect the cell proliferation. After cell seeding, the same steps were repeated 24, 48, 72, and 96h.

### 2.9 Transwell assay

The migration and invasive ability of glioma cells were evaluated and analyzed by Transwell assay. U87MG and U251 glioma cells (5 × 10^4^ cells) were placed into the chambers without Matrigel coating for migration analysis and to simulate an invasive environment in the chambers coated with Matrigel for invasion analysis. In the upper chamber, medium without FBS was added. In the lower chamber, a medium containing 10% FBS was added. After 24 hours of incubation, migrating or invading cells were fixed with 4% paraformaldehyde solution and stained with crystal violet. Finally, quantitative analysis.

### 2.10 Relative abundance analysis of tumor-infiltrating immune cells

To assess the correlation between TMEM115 expression and tumor-infiltrating immune cells, we used a CIBERSORT-based core algorithm using markers for 22 immune cells provided on the CIBERSORT website (CIBERSORTx, http://cibersort.stanford.edu/) to calculate TMEM115 expression and immune infiltration of tumor-infiltrating immune cells.

### 2.11 Statistical analysis

T-test and Cox regression were performed to compare the mRNA expression and prognosis of TMEM115 in glioma and non-tumor brain tissues. Experiments involving Western blot analysis and cell function were repeated independently three times. Comparisons between two groups were performed using unpaired or paired two-tailed t-tests, and the significance of differences between more than two groups was analyzed by Two-way ANOVA. The optimal cut-off point for TMEM115 protein expression was determined based on patients’ OS using X-tile (Yale University, USA) (22). Pearson’s test was used to determine TMEM115’s association with immune markers. TMEM115 relationship with clinicopathologic features was assessed using χ^2^ test test. The Cox proportional hazard model was also utilized to identify prognostic factors for TMEM115. Statistical analyses and data visualization were conducted using SPSS (Version: 22.0, IBM, USA) and GraphPad Prism software version 9.0.0 (GraphPad Software, La Jolla, CA), and significance was set at *P* < 0.05.

## 3 Results

### 3.1 TMEM115 mRNA expression, prognostic, and association between clinicopathological characteristics in glioma

**Figure 1:**
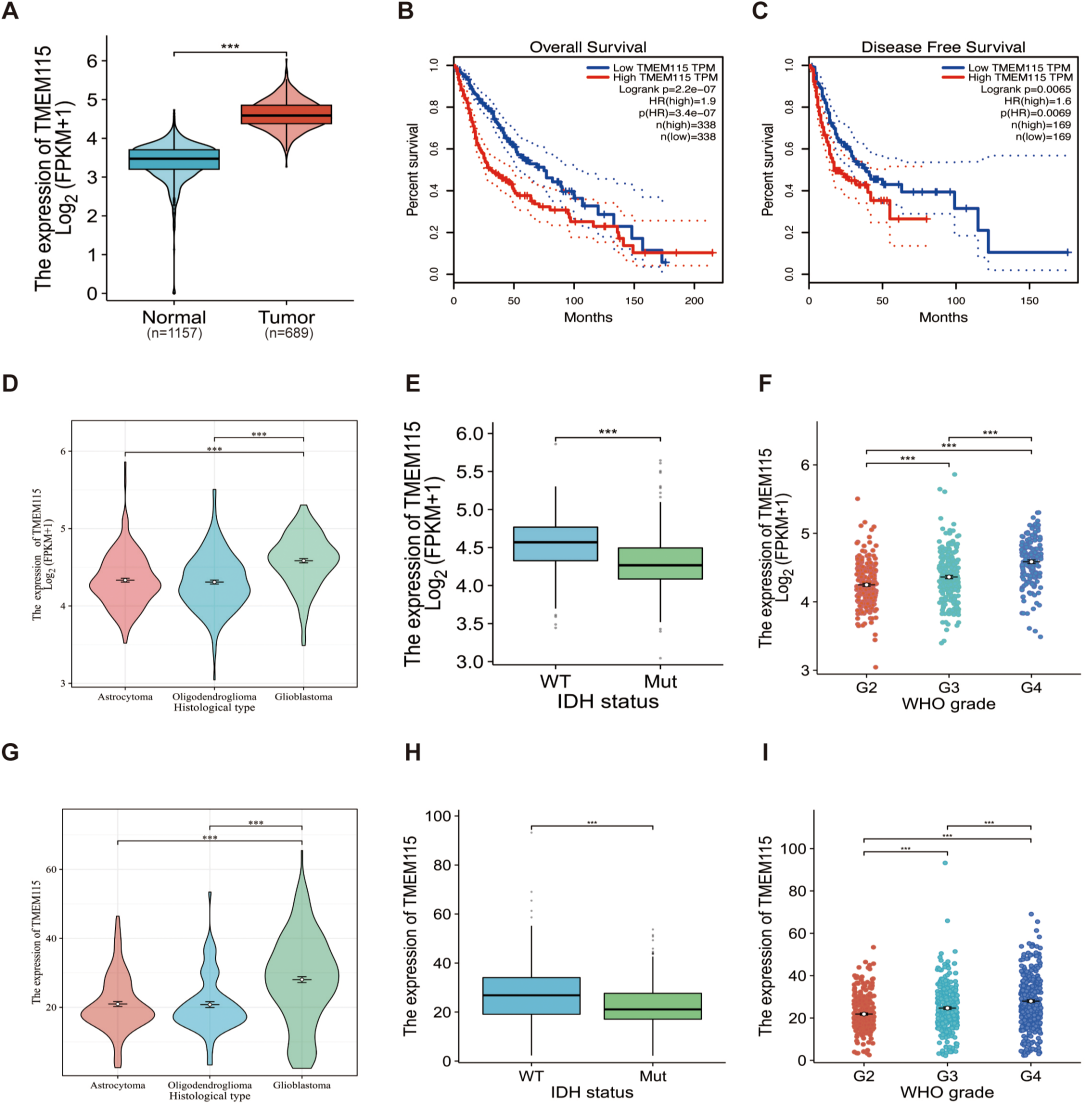
TMEM115 mRNA differential expression in glioma and non-tumoral tissues, prognostic survival analysis, and the association between clinicopathological characteristics in glioma. **(A)** Differential expression of TMEM115 mRNA in glioma (red) (n = 689) compared to non-tumor tissue (blue) (n = 1157) (*P* < 0.05). **(B-C)** OS and DFS survival curve of TMEM115 mRNA in high (red line) and low expression groups (blue line) (HR = 1.9, *P* < 0.05; HR = 1.6, *P* < 0.05). **(D, G)** TMEM115 was prominently raised in glioblastoma according to the 2021 CNS tumor classification (TCGA, Astrocytoma, n = 193, Oligodendroglioma, n = 191, Glioblastoma, n = 160) (CGGA, Astrocytoma, n = 131, Oligodendroglioma, n = 93, Glioblastoma, n = 225) (*P* < 0.05). **(E, H)** TMEM115 was a noteworthy increase in IDH wild-type gliomas in the TCGA database and CGGA 325 dataset, 693 datasets (TCGA, WT, n = 246, Mut, n = 443) (CGGA, WT, n = 435, Mut, n = 531) (*P* < 0.05). **(F, I)** TMEM115 was significantly increased in high-grade gliomas in the TCGA database and the CGGA 325 dataset, 693 datasets (TCGA, G2, n = 224, G3, n = 245, G4, n = 168) (CGGA, G2, n = 291, G3, n = 334, G4, n = 388) (*P* < 0.05).

We first assessed whether TMEM115 is a marker in glioma. The results showed a significant increase in TMEM115 mRNA expression in glioma tissues compared to nonmalignant brain tissues (*P* < 0.05) (**Figure 1A**). To further determine the potential of TMEM115 as a prognostic biomarker for gliomas, correlation analysis with OS and DFS was performed. In glioma patients, elevated TMEM115 expression was strongly correlated with adverse prognosis (HR = 1.9, *P* < 0.05; HR = 1.6, *P* < 0.05) (**Figures 1B, C**). Furthermore, given the new definition of adult gliomas in the 2021 WHO classification, we investigated the expression of TMEM115 in IDH mutation astrocytoma, IDH mutation, and 1p19q codeletion oligodendroglioma, and IDH wild-type glioblastoma (*P* < 0.05) (**Figure 1D**). The significant upregulation of TMEM115 expression in IDH wild-type glioblastoma. In addition, differences in TMEM115 between other clinical variable groupings (IDH status, WHO grade) were directly analyzed based on the TCGA database (*P* < 0.05) (**Figures 1E, F**). These results were also validated in the CGGA database (**Figures 1G-I**). These suggest the clinical significance of TMEM115 and its potential utility as a prognostic biomarker for glioma patients.

### 3.2 TMEM115 expression, localization, prognostic potential in glioma tissues, and relationship with clinical features

**Figure 2:**
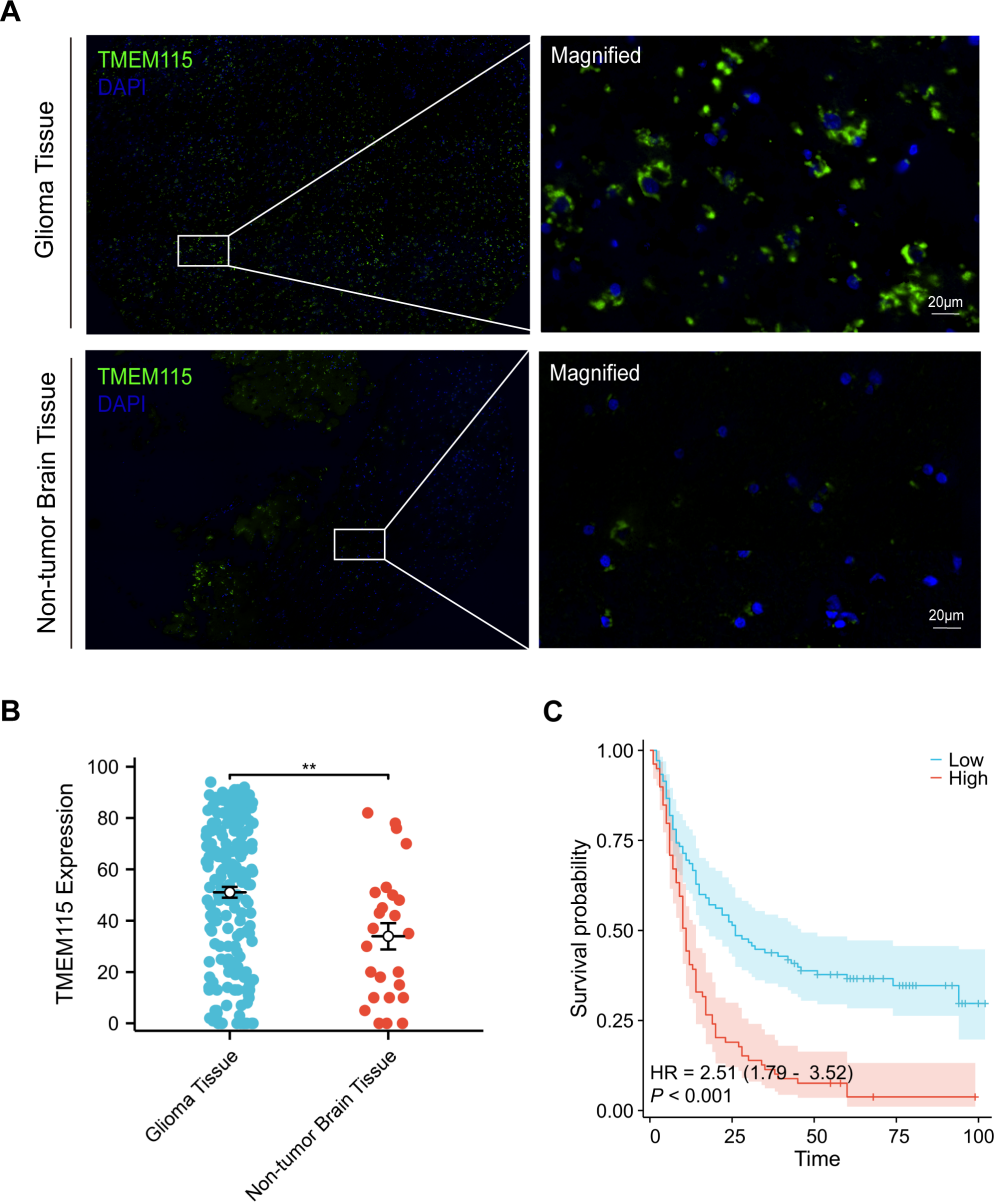
TMEM115 protein differential expression in glioma and non-tumoral tissues, prognostic survival analysis, and the association between clinicopathological characteristics in glioma. **(A)** Representative images demonstrating TMEM115 protein expression and immunostaining in glioma and non-tumor brain tissues. TMEM115 (green) and DAPI (blue). Scale bar, 20 μm. **(B)** Quantitative dot plot illustrating TMEM115 protein expression in glioma (n = 189) (blue) and non-tumor brain tissue (n = 25) (red), **P < 0.01. **(C)** Survival curve demonstrating that patients with enhanced TMEM115 expression (red line, High, n = 80) have significantly poorer prognosis compared to those with decreased TMEM115 expression (blue line, Low, n = 109) (*P* < 0.001).

Given the limitations of predicting protein expression only based on transcription levels (16), we conducted mIHC staining of glioma TMA sections to assess TMEM115 protein expression and localization. TMEM115 protein was found to be primarily localized in glioma cells plasma membrane (**Figure 2A**). Consistent with mRNA levels, elevated levels of TMEM115 were observed in tumor tissues (**Figures 2A, B**).

The optimal cut-off value for TMEM115 protein expression in gliomas was determined based on the OS of glioma patients and categorized into high and low-expression groups. TMEM115 protein levels were classified as a low and high-expression group based on a threshold of 64. Chi-square test results revealed a significant correlation between TMEM115 protein expression in tumor cells and WHO grading (χ^2^ = 17.134, *P* < 0.001), but no significant associations were found with gender, age, histological classification, molecular typing, or the presence of radiotherapy and chemotherapy (**Table 1**).

**Table 1:** Correlation of TMEM115 protein expression with clinicopathological characteristics in glioma patients.

TMEM115 expression					
Characteristic	n	Low	High	Pearson χ^2^	*P*
Total	189	109(57.7)	80(42.3)		
Gender				0.084	0.772
Male	104	59(56.7)	45(43.3)		
Female	85	50(58.8)	35(41.2)		
Age				2.039	0.153
≤60	122	75(61.5)	47(38.5)		
>60	67	34(50.7)	33(49.3)		
Histological classification				4.202	0.240
a	135	73(54.1)	62(45.9)		
B	19	14(73.7)	5(26.3)		
c	26	15(57.7)	11(42.3)		
d&e	9	7(77.8)	2(22.2)		
Molecular classification				0.000	0.991
IDH1^R132H/mut^	138	79(57.2)	59(42.8)		
IDH1^R132H/WT^	35	20(57.1)	15(42.9)		
WHO Grade				17.134	<0.001*
1&2	38	32(84.2)	6(15.8)		
3	42	26(61.9)	16(38.1)		
4	109	51(46.8)	58(53.2)		
Chemotherapy				0.028	0.868
TMZ	22	13(59.1)	9(40.9)		
None	166	95(57.2)	71(42.8)		
Radiotherapy				0.463	0.496
Yes	40	21(52.5)	19(47.5)		
No	147	86(58.5)	61(41.5)		

*p<0.05.

a, Astrocytoma, IDH1^R132H^mutant; b, Oligodendroglioma, IDH1^R132H^mutant; c, GBM, IDH1^R132H^wt; d, Pilocytic astrocytoma; e, Pleomorphic xanthoastrocytoma.

In the univariate analysis, TMEM115 protein expression (HR = 2.450, *P* < 0.001), age (HR = 2.261, *P* < 0.001), gender (HR = 1.588, *P* = 0.008), histologic classification (HR = 0.796, *P* = 0.024) and grade (HR = 2.462, *P* < 0.001) were found to significantly impact patient survival. In the multivariate analysis, TMEM115 protein expression (HR = 1.840, *P* = 0.001), age (HR = 1.522, *P* = 0.019), and grade (HR = 2.066, *P* < 0.001) were independently associated with 5-year OS in glioma patients (**Table 2**). Survival analyses demonstrated that high TMEM115 levels led to poorer OS in glioma patients (HR = 2.51, *P* < 0.001) (**Figure 2C**). Collectively, these findings support TMEM115 as a potential indicator for glioma patient survival.

**Table 2:** Univariate and multivariate analysis of prognostic factors for 5-year survival in glioma patients.

	Univariate analysis				Multivariate analysis			
	HR	*P* >|z|	95% CI		HR	*P* >|z|	95% CI	
TMEM115 expression	2.450	<0.001*	1.747	3.438	1.840	0.001*	1.308	2.611
Age (y) ≤ 60 vs > 60	2.261	<0.001*	1.613	3.171	1.522	0.019*	1.072	2.161
Gender Male vs Female	1.588	0.008*	1.131	2.229	1.353	0.087	0.958	1.911
Histological classification a vs b vs c vs d&e	0.796	0.024*	0.653	0.971	0.934	0.519	0.758	1.150
Molecular classification IDH1^R132H/mut^ vs IDH1^R132H/WT^	0.900	0.625	0.591	1.371				
Grade 1&2 vs 3 vs 4	2.462	<0.001*	1.900	3.189	2.066	<0.001*	1.564	2.728
Chemotherapy Yes vs No	0.776	0.341	0.461	1.307				
Radiotherapy Yes vs No	0.735	0.140	0.488	1.106				

*p<0.05.

a, Astrocytoma, IDH1^R132H^mutant; b, Oligodendroglioma, IDH1^R132H^mutant; c, GBM, IDH1^R132H^wt; d, Pilocytic astrocytoma; e, Pleomorphic xanthoastrocytoma.

### 3.3 TMEM115 high expression accelerated malignant phenotype of glioma cells

**Figure 3:**
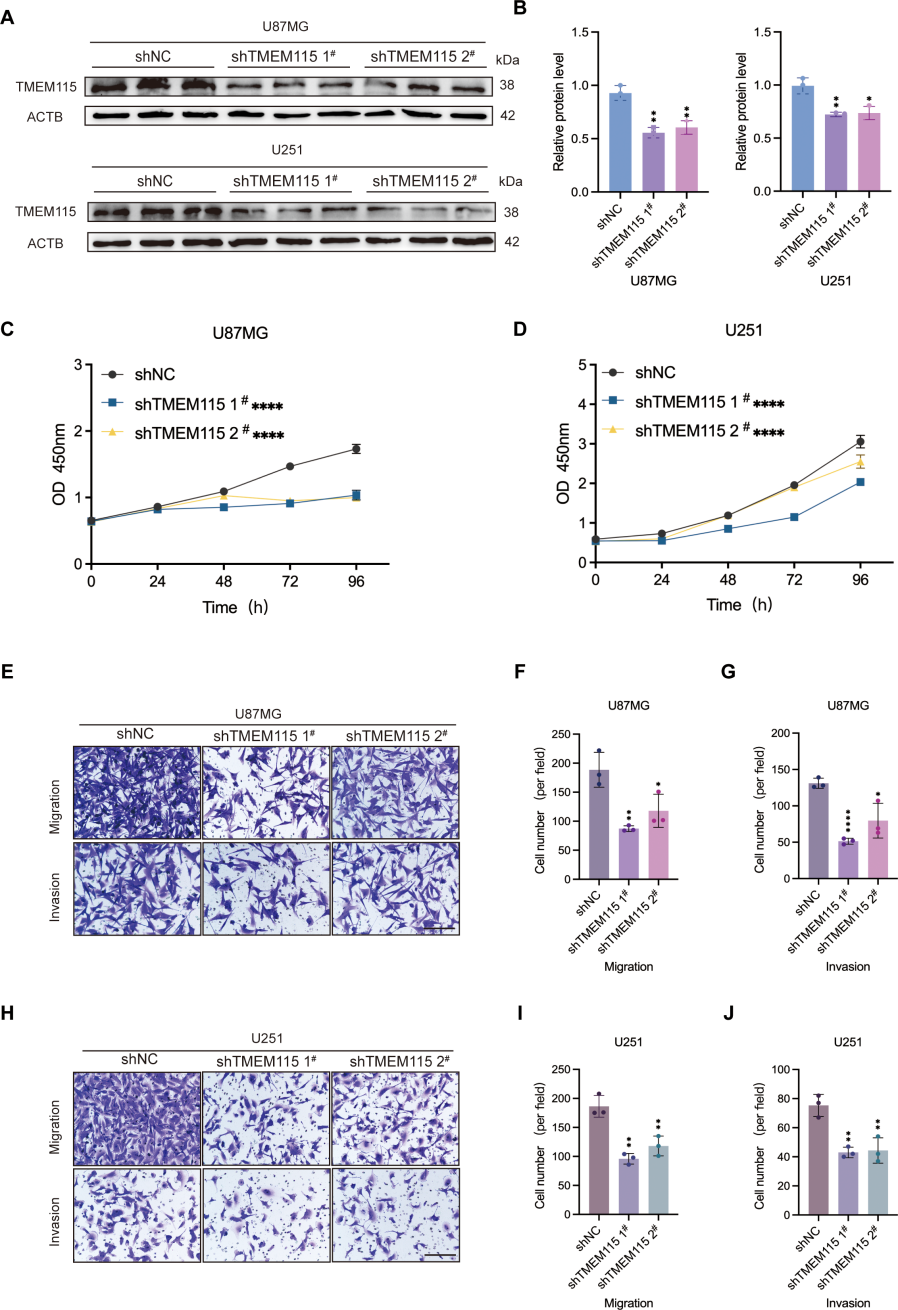
High expression TMEM115 accelerated malignant phenotype of glioma cells. **(A, B)** Knockdown of TMEM115 in U87MG and U251 glioma cells and validation of knockdown efficiency. **(C, D)** Cell proliferation after knockdown of TMEM115 was assessed by CCK-8 assay. **(E-J)** The ability to migrate and invade was measured using the Transwell assay after knocking down TMEM115. *P < 0.05, **P < 0.01, ****P < 0.0001, Scale bar = 50 μm.

To elucidate the effect of TMEM115 on glioma phenotype and to determine its oncogenic role, we designed and established shRNAs and their respective lentiviral systems. The U87MG and U251 cell lines, which are relatively malignant, were selected to be infected with shRNAs, firstly, using lentiviral infection to generate stable knockdown cell lines, thus promoting the knockdown of TMEM115. Knockdown of TMEM115 significantly reduced the protein expression of glioma cells compared to those infected with negative controls (**Figures 3A, B**) The results of the CCK-8 assay showed that the proliferative capacity of U87MG and U251 cells was significantly decreased with the reduction of TMEM115 levels (**Figures 3C, D**) Subsequently, using Transwell assay assessment, after TMEM115 downregulation, the migration and invasion abilities of U87MG and U251 glioma cells were inhibited (**Figures 3E-J**). Thus, the effects of TMEM115 on glioma proliferation, migration, and invasion were demonstrated.

### 3.4 TMEM115 significant correlations with the abundance of TIICs and immune checkpoints in glioma

**Figure 4:**
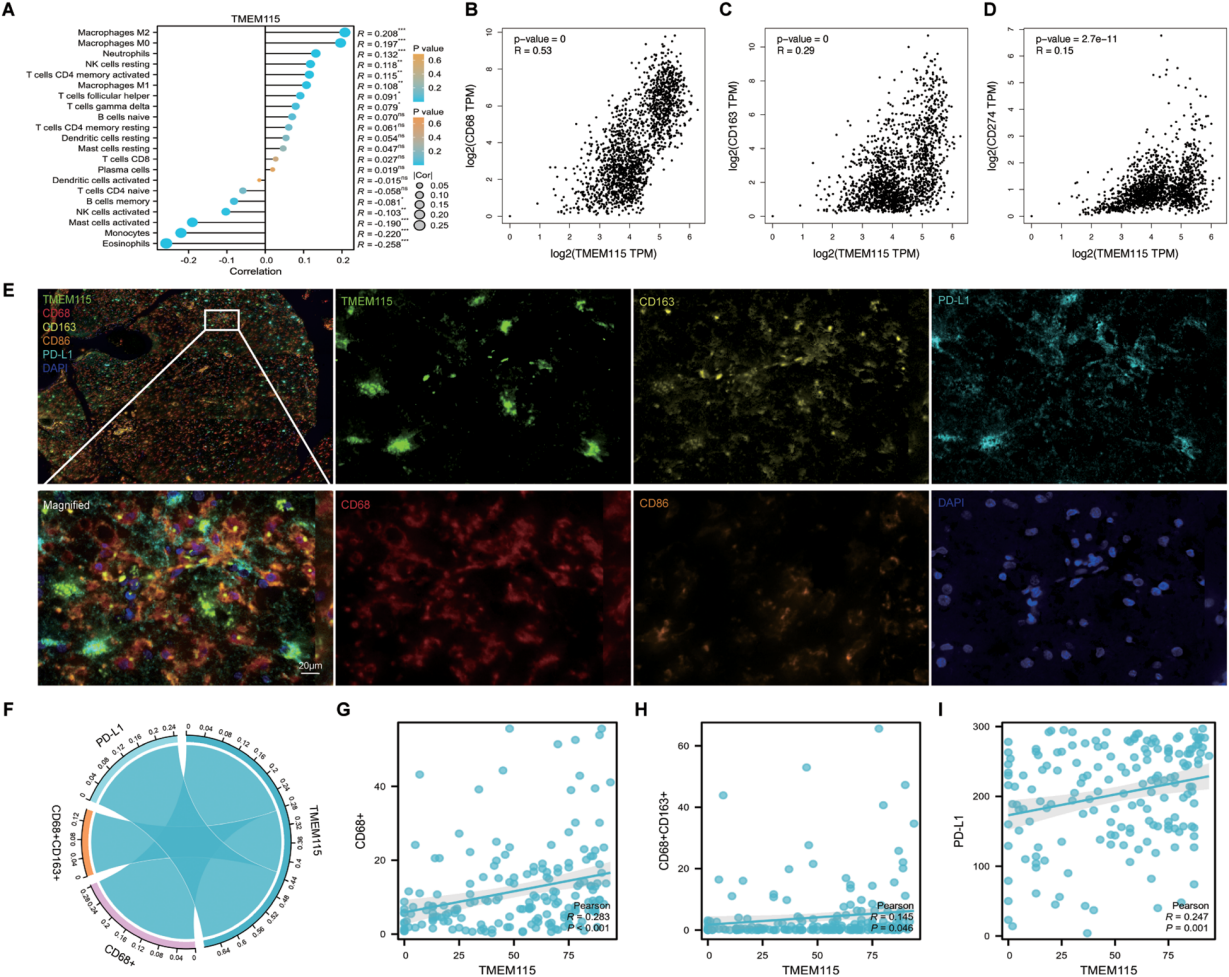
TMEM115 expression and its correlation with TIICs and immune checkpoints in glioma. **(A)** CIBERSORT analysis of 22 infiltrating immune cell types associated with TMEM115 in glioma. **(B-D)** Based on TCGA and GTEx databases analyzed the correlation of TMEM115 mRNA expression in gliomas with CD68 (R = 0.53, *P* < 0.001), CD163 (R = 0.29, *P* < 0.001) and common immune checkpoint CD274 (R = 0.15, *P* = 2.7e-11). **(E)** Representative images demonstrating TMEM115 protein expression and immunostaining in glioma tissues. Incorporate monochrome and overlapping images. TMEM115 (green), CD68 (red), CD163 (yellow), CD86 (orange), PD-L1 (lake blue), and DAPI (blue). Scale bar, 20 μm. **(F)** Chord plots depicting the relationship between TMEM115 expression with M2 macrophages, and PD-L1 in glioma tissues. **(G-I)** Correlation analysis between TMEM115 and CD68^+^ (R = 0.283, *P* < 0.001), CD68^+^CD163^+^ (R = 0.145, *P* = 0.046) and PD-L1 (R = 0.247, *P* = 0.001) in glioma tissue.

Considering that TME plays a crucial role in determining the response of gliomas to immunotherapy, we extracted RNA-seq data from gliomas in TCGA. We evaluated the correlation between TMEM115 expression and TIICs. The results showed that TMEM115 was positively correlated with M2 macrophages (**Figure 4A**). Additionally, we confirmed this result by incorporating clinical information from the GTEx databases based on the TCGA database. Since immune checkpoints can fuel tumor immunotherapy, we also evaluated the correlation of TMEM115 with the common immune checkpoints, the results showed that TMEM115 was correlations with CD274 (PD-L1) (R = 0.15, *P* = 2.7e-11) (**Figures 4B-D**). To verify this, we performed mIHC staining and analyzed the expression of TMEM115 protein in the glioma TME using Pearson’s test (**Figure 4E**). We found that TMEM115 protein levels in tumor tissues were positively associated with CD68^+^ (R = 0.283, *P* < 0.001) and CD68^+^CD163^+^ (R = 0.145, *P* = 0.046) macrophages (**Figures 4F, G**). And interestingly, protein expression also correlated with the immune checkpoint PD-L1 (R = 0.247, *P* = 0.001) (**Figure 4I**).

## 4 Discussion

We used bioinformatics to explore the association between TMEM115 mRNA expression, glioma patients’ prognosis, and its relationship with clinicopathologic features. Upon validation, we examined the effect of TMEM115 on glioma cell phenotype. Furthermore, we established a correlation between TMEM115 protein levels and the degree of glioma malignancy and TME using mIHC. Further analysis confirmed TMEM115 levels as a potential indicator for assessing glioma patient survival.

Gliomas present considerable challenges to conventional treatment modalities due to their unique environment, including the blood-brain barrier and intricate tumor immune responses (23). Investigations into the association between TMEM115 protein and human diseases revealed that TMEM115 may indirectly influence protein glycosylation in the Golgi (24). Altered glycosylation is often regarded as a hallmark of cancer (25, 26). It can promote glioma growth and temozolomide resistance (27), and also promotes the dissociation of hexokinase 2, a key metabolic enzyme that catalyzes the glycolytic pathway, from the mitochondria by highly activating aerobic glycolysis and binds to an inhibitor of NF-κB, which promotes the expression of PD-L1 and contributes to immune escape from the tumor (28). Hence, our present research was initiated to determine whether TMEM115 could serve as a promising biomarker for glioma treatment. As anticipated, TMEM115 mRNA levels were found to be elevated in tumor than non-tumor samples. Moreover, mIHC results demonstrated predominant TMEM115 protein expression on the plasma membrane of both glioma and benign non-glioma cells, with varying degrees of expression. Importantly, the protein levels were consistent with mRNA expression, exhibiting significantly increased expression in tumor tissues with high TMEM115 expression. Additionally, the proliferation, migration, and invasion of glioma cells were decreased when TMEM115 expression was downregulated.

The TME is a complex environment comprising various components, such as immune cells, vasculature and signaling molecules, where tumor cells thrive (29). Understanding the TME is essential for developing effective immunotherapy strategies, particularly considering the role of tumor immune-infiltrating cells and immune checkpoints in driving immunosuppression (30, 31). In this study, we investigated the relationship between TMEM115 protein expression and TAMs as well as immune checkpoints in glioma TMAs. Our findings revealed a positive correlation between TMEM115 protein expression and CD68^+^ and CD68^+^CD163^+^ (M2 macrophages), which are associated with poor prognosis in many solid tumors (32), aligning with previous research across various solid tumors, thereby suggesting a consistent role of TMEM115 in tumor progression. We hypothesized that TMEM115 and M2 macrophages promote glioma cell growth and influence their biological behavior. To further elucidate this mechanism, primary macrophages can be isolated and cultured for differentiation analysis using flow cytometry. In the realm of tumor immunotherapy, anti-PD-L1 antibodies demonstrate significant potential, offering new hope to cancer patients (33). Our study revealed a positive correlation between TMEM115 mRNA expression and PD-L1, with TMEM115 protein expression was coincided with mRNA levels. This finding provides novel insights into the sensitivity of anti-PD-L1 antibodies. At the protein level, TMEM115 may participate in PD-L1 post-translational modifications, such as glycosylation, which can influence PD-L1 stability and membrane localization, thereby enhancing its binding to PD-1. PD-L1 can bind to PD-1 on T cells, inhibits T cell function and promotes tumor immune evasion (34). Anti-PD-L1 antibodies can block this interaction, restoring T cell anti-tumor activity (35). The TMEM115 and PD-L1 correlation suggests TMEM115 overexpressing tumors may be more drug sensitivity to anti-PD-L1 therapy. This is because high TMEM115 and PD-L1 expression implies tumor reliance on the PD-L1 pathway for immune evasion. In summary, we propose that elevated the expression of TMEM115 may facilitate immune evasion via two potential mechanisms. Initially, through glycosylation - dependent M2 macrophage polarization: TMEM115 might regulate the glycosylation status of key membrane proteins, thereby enhancing the synthesis and secretion of immunosuppressive factors like IL - 10 and TGF - β. This process could drive the differentiation of monocytes into M2 - TAMs), consequently intensifying the immunosuppressive microenvironment. Secondly, regarding the metabolic - immunoregulation of PD - L1: glycosylation modifications may boost PD - L1 expression within the lactic acid microenvironment arising from the Warburg effect. Hence, for individuals exhibiting high TMEM115 expression, a combination therapy using a PD - L1 inhibitor along with a glycosylation inhibitor (such as a Golgi function modifier) targeting TMEM115 is anticipated to yield a synergistic therapeutic benefit.

To bridge the gap between laboratory findings and clinical application, we also examined the association between TMEM115 and 5-year OS rates along with clinical features in our study. We observed a correlation between TMEM115 and WHO grade, with elevated expression linked to a poorer prognosis, leading us to hypothesize that TMEM115 potentially plays a role in glioma progression, thereby influencing tumor growth. These findings suggest that TMEM115 is an oncogene driving glioma progression and a potential predictor of patient survival.

The present study had several limitations. First, further evidence of the role of TMEM115 in glioma progression and macrophage polarization using animal models is needed. Second, the mechanism of whether TMEM115 expression drives glycosylation and affects PD-L1 binding or immune responses remains to be elucidated. Third, the correlation with clinical outcomes lacks a mechanistic explanation and the current dataset is cross-sectional, lacking longitudinal data. In summary, TMEM115 could be a promising prognostic marker and a potential target for glioma immunotherapy. Future research endeavors ought to be directed toward confirming the functional mechanisms of TMEM115 in gliomas and delving deeper into its therapeutic potential within the realm of immunotherapy. The ultimate objective is to contribute to the emergence of innovative clinical treatment strategies for gliomas.

## 5 Conclusions

In summary, this is a report of TMEM115 expression in glioma patients. TMEM115 was found to be highly expressed in glioma tissues and to affect the prognostic survival of glioma patients, influencing the progression of gliomas. In addition, these results elucidate the relationship between TMEM115 expression and the expression of macrophages and common immune checkpoints in gliomas. Promoting the understanding of clinical treatment of glioma warrants further research in the future.

## Data Availability

The original contributions presented in the study are included in the article/**Supplementary Material**. Further inquiries can be directed to the corresponding author.
