# Supplementary figures and images for: TMEM115: a promising marker for glioma immunotherapy and prognosis
Source: Front Immunol. 2025 Jun 9;16:1598499. doi: 10.3389/fimmu.2025.1598499 (PMC12183186; doi:10.3389/fimmu.2025.1598499)

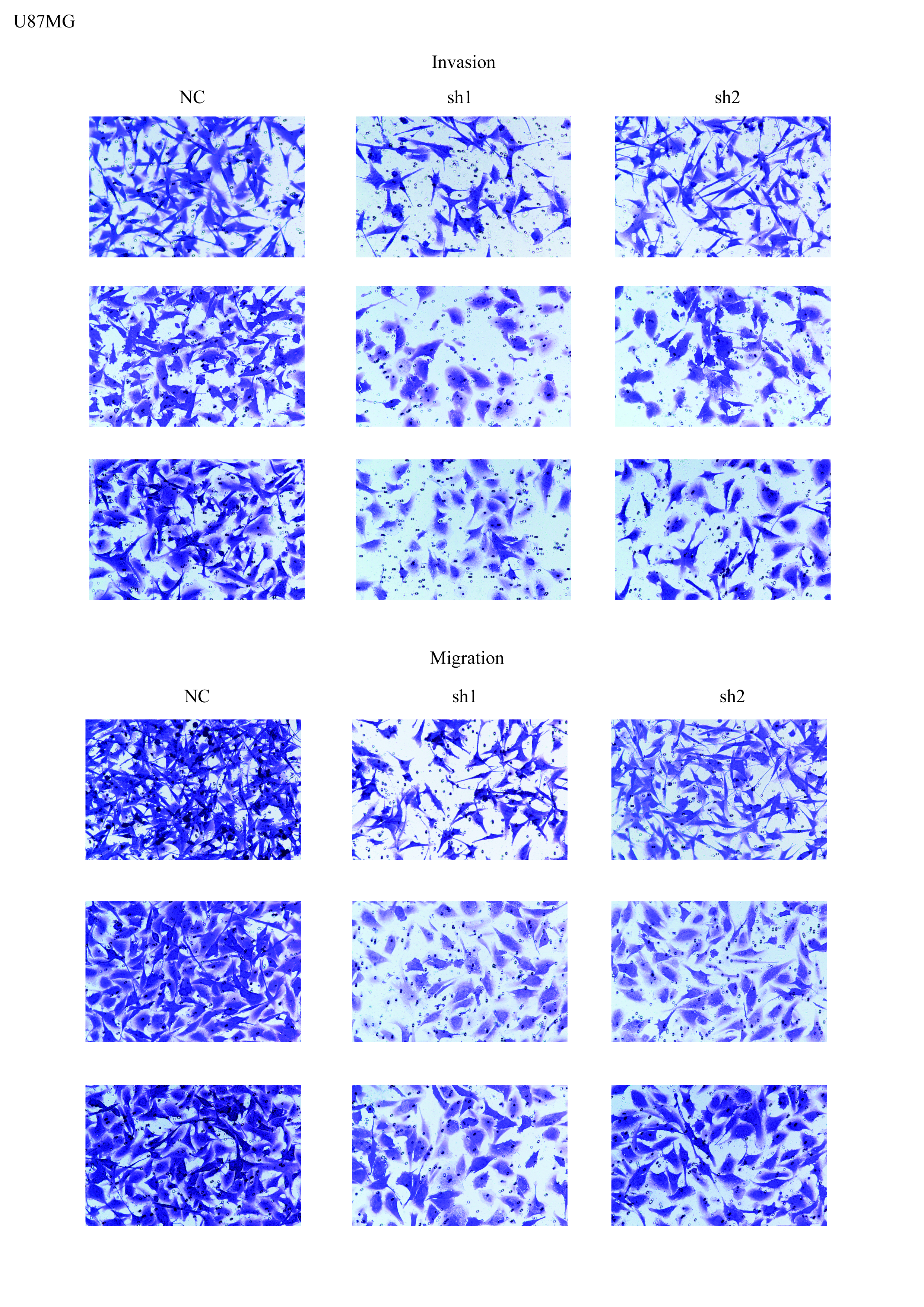

Supplement: Supplementary file 1 [file Image1.tif]

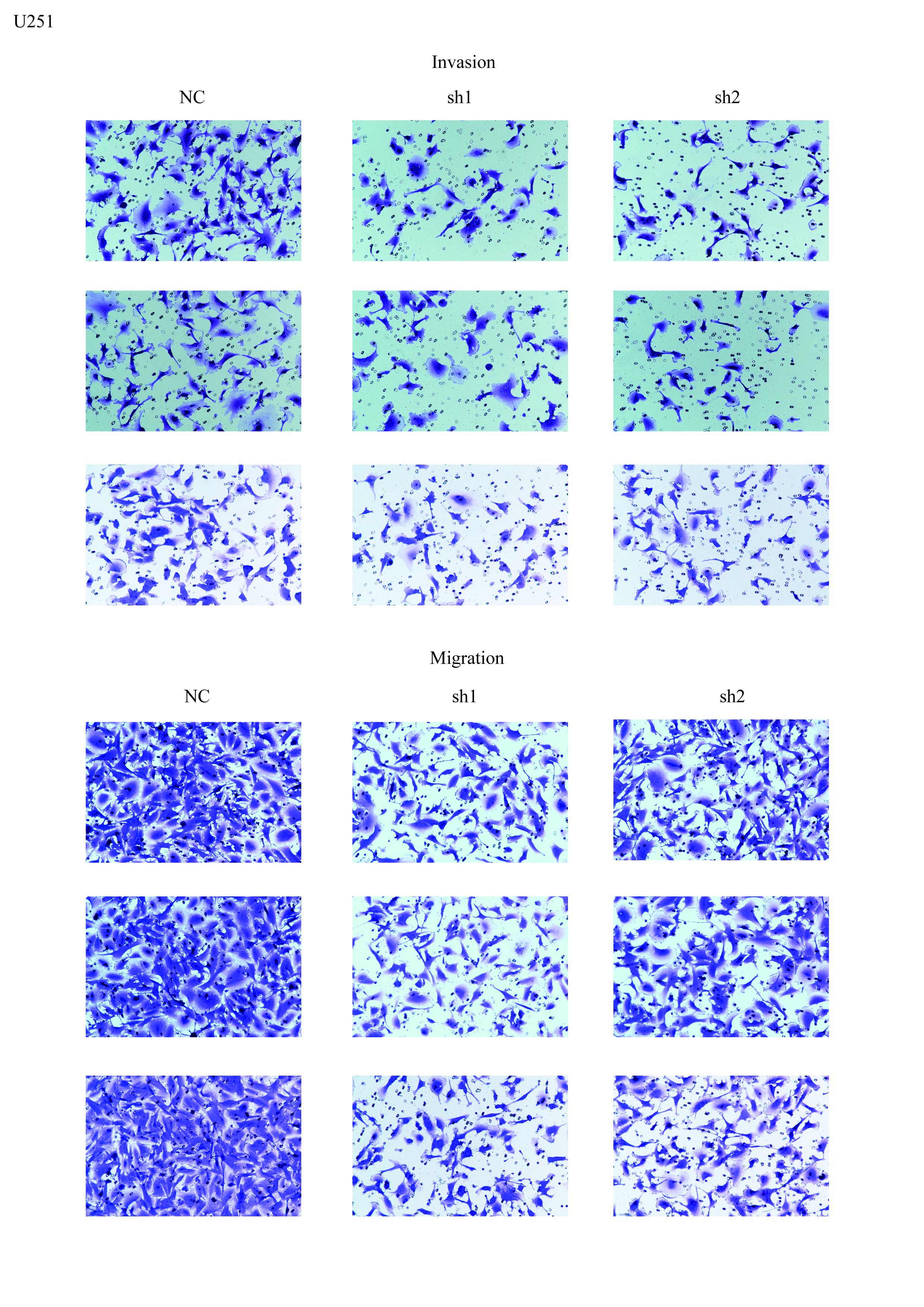

Supplement: Supplementary file 2 [file Image2.tif]

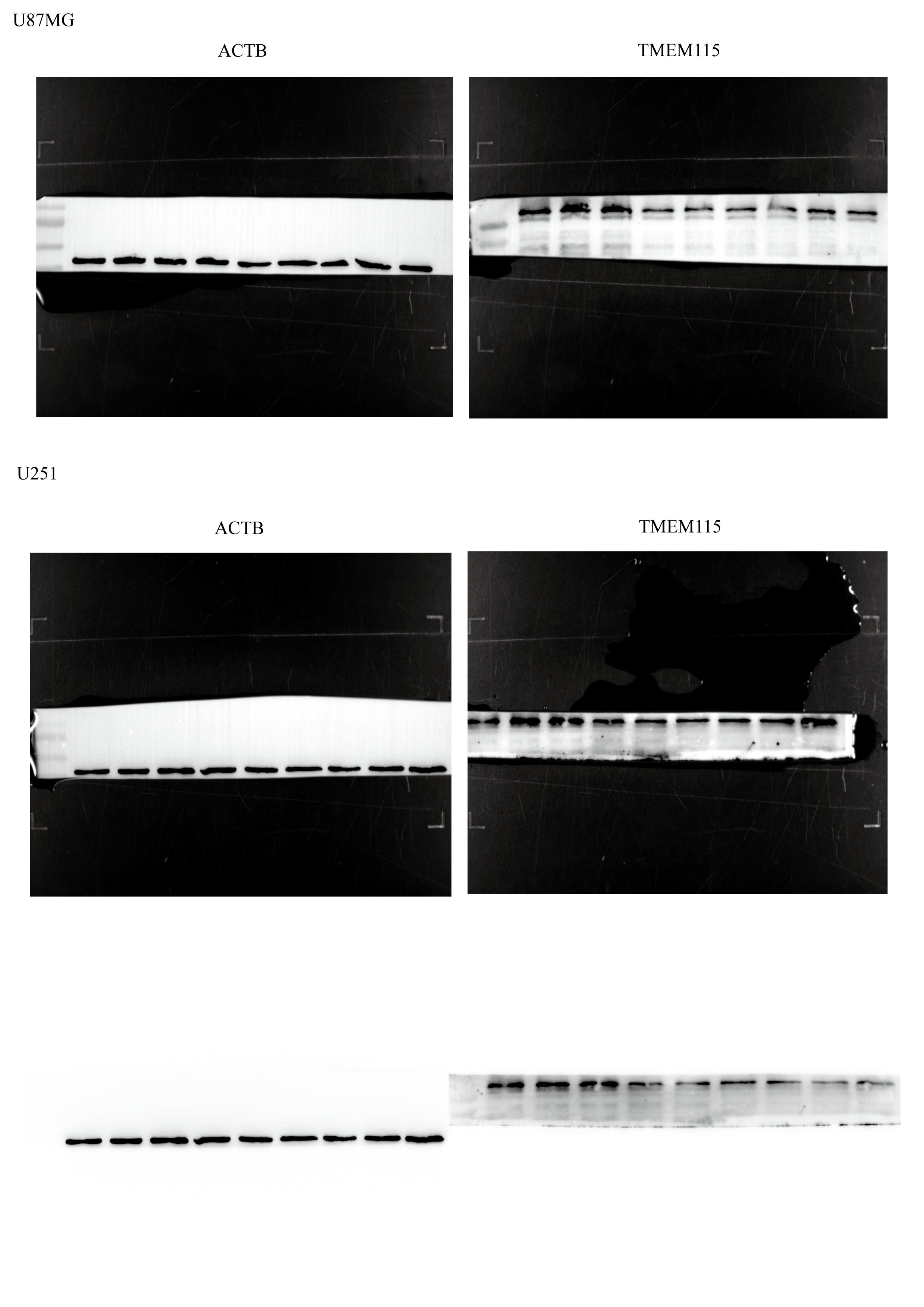

Supplement: Supplementary file 3 [file Image3.tif]
